# Heritability of biting time behaviours in the major African malaria vector *Anopheles arabiensis*

**DOI:** 10.1186/s12936-023-04671-7

**Published:** 2023-08-16

**Authors:** Nicodem J. Govella, Paul C. D. Johnson, Gerry F. Killeen, Heather M. Ferguson

**Affiliations:** 1 Environmental Health and Ecological Sciences Department, Ifakara Health Institute, Dar es Salaam, Tanzania; 2 Institute of Biodiversity, Animal Health and Comparative Medicine, University of Glasgow, Glasgow, G12 8QQ UK; 3 School of Life Sciences and Bioengineering, Nelson Mandela African Institution of Science and Technology, Arusha, Tanzania; 4 Department of Vector Biology, Liverpool School of Tropical Medicine, Pembroke Place, Liverpool, L3 5QA UK; 5 School of Biological, Earth & Environmental Sciences and Environmental Research Institute, University College Cork, Cork, T23 N73K Republic of Ireland

**Keywords:** Feeding behaviour, Malaria vector, Heritability, Phenotypic plasticity, Anopheles arabiensis

## Abstract

**Background:**

The use of insecticide-treated nets for malaria control has been associated with shifts in mosquito vector feeding behaviour including earlier and outdoor biting on humans. The relative contribution of phenotypic plasticity and heritability to these behavioural shifts is unknown. Elucidation of the mechanisms behind these shifts is crucial for anticipating impacts on vector control.

**Methods:**

A novel portable semi-field system (PSFS) was used to experimentally measure heritability of biting time in the malaria vector *Anopheles arabiensis* in Tanzania. Wild *An. arabiensis* from hourly collections using the human landing catch (HLC) method were grouped into one of 3 categories based on their time of capture: early (18:00–21:00), mid (22:00–04:00), and late (05:00–07:00) biting, and placed in separate holding cages. Mosquitoes were then provided with a blood meal for egg production and formation of first filial generation (F1). The F1 generation of each biting time phenotype category was reared separately, and blood fed at the same time as their mothers were captured host-seeking. The resultant eggs were used to generate the F2 generation for use in heritability assays. Heritability was assessed by releasing F2 *An. arabiensis* into the PSFS, recording their biting time during a human landing catch and comparing it to that of their F0 grandmothers.

**Results:**

In PSFS assays, the biting time of F2 offspring (early: 18:00–21:00, mid: 22:00–04:00 or late: 05:00–07:00) was significantly positively associated with that of their wild-caught F0 grandmothers, corresponding to an estimated heritability of 0.110 (95% CI 0.003, 0.208). F2 from early-biting F0 were more likely to bite early than F2 from mid or late-biting F0. Similarly, the probability of biting late was higher in F2 derived from mid and late-biting F0 than from early-biting F0.

**Conclusions:**

Despite modest heritability, our results suggest that some of the variation in biting time is attributable to additive genetic variation. Selection can, therefore, act efficiently on mosquito biting times, highlighting the need for control methods that target early and outdoor biting mosquitoes.

**Supplementary Information:**

The online version contains supplementary material available at 10.1186/s12936-023-04671-7.

## Background

The most important current malaria control interventions are insecticide-treated nets (ITNs) and indoor residual spraying (IRS) [1, 2]. These tools have averted more than 600 million clinical cases in Africa since 2000 [1]. The success of these interventions derives from their ability to exploit key aspects of the biting and resting behaviour of African mosquito vectors, including their propensity to bite humans indoors during sleeping hours and rest indoors after feeding [3, 4]. The most important malaria vector species in Africa, the *Anopheles gambiae* species complex [4, 5] and *Anopheles funestus* [4, 5], typically exhibit these behaviours [6, 7]. Despite the success of ITNs and IRS, their effectiveness is being undermined by mosquito adaptations that allow resistance or evasion from such interventions. Most notable is the widespread emergence of insecticide resistance [8]. Additionally, there is growing evidence of changes in vector behaviour in Africa and elsewhere [9–12] that allow vectors to reduce contact with ITNs and IRS [7]. While the molecular and genetic basis of insecticide resistance [13, 14] and its impact on malaria transmission has been widely investigated [8, 15], much less is known about the basis of mosquito biting behaviour adaptations [16, 17] and their implications for vector control [3, 7].

Mosquito behavioural changes associated with ITNs and IRS include early-exiting from sprayed houses [18], increased outdoor biting at dawn or dusk when people are not protected by ITNs [10, 19–21], and increased feeding on livestock instead of people [22, 23]. The capacity to mount such behavioural adaptations may vary between vector species. For example, historically, *An. gambiae* in East Africa has been reported to feed almost exclusively on people [24], inside houses, and late at night [4, 6], while its sibling species, *Anopheles arabiensis,* feeds more flexibly on humans and cattle [25, 26], indoors or outdoors, [20, 27], often in the early evening and at dawn [20, 28]. With the wide-use of ITNs, the relative abundance of *An. gambiae* compared to *An. arabiensis* has plummeted in several settings [29, 30] due to high propensity of this species to feeding indoors and late at night. The timing of this behaviour coincides with when the majority of people are indoors and under ITNs, thus increases the risk of *An. gambiae* having fatal contact with insecticides. In west Africa, there are reports of a change towards early evening or early morning biting, and more outdoor biting in *Anopheles coluzzii* [31] and *An. funestus* [11, 19]. Similarly, the proportion of “early” (18:00–21:00 h) and outdoor biting by the malaria vector *Anopheles farauti* in the southwest Pacific increased after the implementation of IRS [10, 32]. There is also evidence of shifts in host choice from humans to cattle in African malaria vectors following ITN and IRS implementation [22, 23].

Prediction of the impact of mosquito behavioural changes on vector control requires understanding of the underlying mechanisms for the changes. It is unknown whether behavioural shifts reflect evolutionary adaptations in response to selection by ITNs/IRS, or are manifestations of pre-existing phenotypic plasticity. These possibilities have different implications for control. The first hypothesis, referred to as true *behavioural resistance*, is that behavioural traits are evolving in response to selection from interventions. Behavioural resistance traits could thus spread and become fixed in populations [3]. The second hypothesis, referred to as *behavioural resilience* [9], is that vector species were always capable of expressing alternative biting phenotypes with this plasticity only exhibited in response to environmental variation that reduces human host availability. Here biting behaviour may rapidly revert to baseline phenotype when control interventions are lifted [16]. Behavioural resilience may define the limits of immediate behavioural responses to interventions [3], whereas behavioural resistance implies that vectors can increasingly adapt their biting phenotypes to avoid indoor interventions over time; thus progressively eroding the proportion of exposure that can be prevented by their use. This mechanism may pose a larger problem than plasticity within a fixed range. While it has been observed for many years that use of ITNs is associated with shift in mosquito biting time behaviours, there has been relatively limited investigation of the genetic basis of host-seeking in mosquitoes.

In this study, heritability in the biting time of the major African malaria vector *An. arabiensis* was experimentally investigated using a novel portable semi-field system (PSFS), in Tanzania. Biting time phenotypes of wild-caught mothers (F0) were compared to phenotypes of their offspring (second generation) under controlled conditions.

## Methods

### Study site

All experiments were conducted in Lupiro village (− 8.38 S, 36.67 E) within the Kilombero Valley, an area of moderate to high endemic malaria transmission in south-eastern Tanzania [33]. Currently, *An. arabiensis* is the most abundant malaria vector species in this area [34]. Biting activity in this *An. arabiensis* population can start as early as dusk, with a peak around midnight and smaller peak toward morning [27]. Most residents spend their time outdoors until ~ 10 pm when they go inside homes to sleep; with most reporting use of ITNs [27]. All mosquito behavioural assays were conducted within a bespoke semi-field system, here referred to as a portable semi-field system (PSFS) installed temporarily in Lupiro Village (details in Additional file 1). The PSFS was located in the same village where wild mosquitoes (parental generation) were collected to generate offspring for use in experiments.

### Assays for heritability in biting time

In Lupiro village, host-seeking female *An. arabiensis* were collected at different times of the night using human landing catches (HLC). With HLC, a volunteer sat on a chair while exposing his legs and aspirating mosquitoes that attempted to feed on them. The collections were conducted in the peridomestic area around four houses (Additional file 2**)**. In brief, volunteers collected mosquitoes hourly between 18:00 and 07:00 h. Collections were made for 45 min of each hour leaving 15 min breaks for refreshment and rest for two consecutive nights (14th and 15th of July 2015). Mosquitoes collected each hour were placed in separate paper cups. Mosquitoes were visually identified as belonging to *Anopheles gambiae *sensu lato (*s.l*.) [4, 5] from the hourly collection in the morning after each experimental night, and grouped into one of 3 categories based on their time of capture: early (18:00–21:00), mid (22:00–04:00), and late (05:00–07:00) biting, and placed in separate holding cages. Biting activity was classified into these three discrete categories of unequal length to correspond with times when people are likely to be either indoors and protected by ITNs (mid), or outdoors and unprotected (early and late). Of the 245 female *An. gambiae s.l.* obtained, 218 survived the transition into cages (71, 98, and 49 from early, mid, and late biting groups respectively). These F0 females were provided with a blood meal for egg production as described in Additional file 2. Only eggs from 121 individual mothers (43, 51, and 27 from early, mid and late respectively) were confirmed by PCR to be *An. arabiensis*, and these were used to produce the F1 generation (Additional file 2**)**. These HLC wild caught PCR confirmed *An. arabiensis* females individuals formed the FO parental generation. The eggs from each confirmed *An. arabiensis* mothers were reared separately, transitioning to larvae, and consequently to pupae stage. Pupae reared from each female’s egg clutch were pooled into one of three holding cages according to their mothers’ biting time phenotype. Variation in egg-pupa development rates resulted in the set up of nine F1 cages, three of each biting time phenotype. The F1 generation of each biting time phenotype category were reared separately, and were blood fed in the same time period (early, mid or late) that their mothers were collected in to produce an F2 generation for use in assays within the PSFS (Additional file 2). Due to variations in egg-pupa development rates from F0 to F1, this implies that not all F2 were obtained on the same day, but variable depending on the emergence rates. The F2 were maintained under ambient conditions within the PSFS and on glucose as source of meal until the day of release. Experiments were conducted on the F2 rather than F1 generation to maximize the number of mosquitoes available for experiments.

Heritability was assessed by releasing F2 *An. arabiensis* into the PSFS, recording their biting time during a human landing catch and comparing it to that of their F0 grandmothers. On each night of the experiment, 300 F2 *An. arabiensis* (100 from early, mid, and late F0 phenotype) were released simultaneously into the PSFS at 17:00 h in the evening, with exception of one trial in which only 50 F2 from each of three phenotypes were available (Additional file 2, and Statistical analysis methods below). The age of released mosquitoes ranged from 4 to 10 days old representing a mixture of young and old mosquitoes [35]. Two hours prior to release, F2 female mosquitoes were selected from the cages, placed into the paper cups and marked with either red, yellow, or blue fluorescent dust colors according to their grandmothers’ biting time phenotype (18:00–21:00, 22:00–04:00, and 05:00–07:00). The selection and marking were conducted at 15:00 h on each day of experiment. Mosquitoes were released into the PSFS at 17:00 h. Before recapturing by HLC begun, mosquitoes were left to orient themselves within the PSFS for at least one hour before host entry. A volunteer entered the PSFS at 18:00 h, sat on a chair and commenced HLC (Additional file 2) between 18:00 to 07:00 h. Recapturing was conducted for 45 min in each hour, leaving 15 min break for the volunteer. At the end of each overnight behavioural assay with HLC, the floor, roof and walls within the PSFS were thoroughly searched with a torch to collect any remaining mosquitoes (not caught by HLC) using a Back-pack aspirator. This was to ensure that all mosquitoes used in one experimental night assay were removed before the starting of another experiment. Assays were conducted over 20 nights from 13th August to 1st October 2015. The nightly temperature was measured and collected using a weather station (Additional file 2). Additional HLC collections were conducted at two local houses adjacent to the PSFS (within ~ 40 m) on the same nights as experimental assays to confirm whether the temporal pattern of biting activity observed in the PSFS was consistent with that of the wild population. Wild mosquitoes were collected from inside and outside local houses starting at 18:00 in the evening and end at 07:00 the next morning.

### Data analysis

Before testing for heritability and association in biting time between F0 and F2, the nightly biting profile of F2 offspring on the PSFS and that of wild *An. arabiensis* host-seeking on the same nights as the experiments were compared. This comparison enabled assessment of whether the biting profile of mosquitoes inside the PSFS was representative of natural biting activity. The proportions of mosquitoes caught biting during each time period (early, mid and late) and its’ 95% confidence interval were estimated separately in each location (indoor, outdoor, and semi-field) by fitting logit-binomial generalized linear mixed-effects models (GLMMs) using the *glmer* function of the *lme4 R* package [36], where biting time was modelled as a binomial response (early *vs* mid + late; mid *vs* early + late; late *vs* early + mid) and experimental night (20 nights of replicates) was fitted as an observation-level random effect. Bias in predicted proportions and 95% CIs due to Jensen’s inequality was corrected using the approximation of McCulloch et al*.* [37].

Narrow sense heritability (which describes a fraction of phenotypic variance that can be attributed to variation in the additive effects of genes) of biting time, *h*^*2*^, was estimated as *h*^*2*^ = 2*t*_*F2-F0*_, where *t*_*F2-F0*_ is the correlation between grand-offspring (F2) biting time and grandparental (F0) biting time (see Additional files 3 and 4) for detailed methods and R code for estimation of *h*^*2*^). Owing to an unknown degree of assortative mating within the F1 generation, this *h*^*2*^ estimate is expected to be positively biased, but we show that this bias is likely to be moderate to low (< 17% relative bias) for *h*^*2*^ values below 0.5 and negligible (< 4%) for *h*^*2*^ values below 0.3 (Supplementary Information 3). The correlation coefficient *t*_*F2-F0*_ was estimated by modelling F0 and F2 biting time as an ordered categorical response (early < mid < late) in a mixed-effects ordinal probit GLMM using the *MCMCglmm* package [38]. This approach of modelling a discrete trait as the manifestation of an underlying continuous “liability” (here the tendency towards biting at a specific time) and estimating heritability on the liability scale is standard in quantitative genetics [39]. Random intercepts were included to model inter-batch variation and inter-generational variation within batches, with variances *V*_*b*_ and *V*_*g:b*_ respectively. The correlation between F2 and F0 was estimated as ${t}_{F2-F0}=\frac{{V}_{b}}{{V}_{b}+{V}_{g:b}+1}$. To adjust for potential variation in mean biting time by generation (F0 and F2), temperature, between two HLC volunteers, or over time, the GLMM included fixed effects of generation, temperature (separately for each generation), volunteer, and a natural cubic spline with three degrees of freedom for day of the experiment. Continuous variables (temperature and day) were scaled to have zero mean and unit variance. MCMC was run for 400,000 iterations. MCMC convergence was checked for each parameter by inspection of the trace plots and by requiring that the effective number of samples be greater than 1000. Parameter estimates and 95% credible intervals (CI) were calculated as the mean and 2.5% and 97.5% centiles of the posterior MCMC sample.

In addition to estimating the heritability of biting time, whether individual F2 biting time phenotypes were associated with F0 biting time phenotype was tested. Each F2 biting time phenotype was modelled as a binary response (early *vs* mid + late; mid *vs* early + late; late *vs* early + mid) in a logit-binomial GLMM using the *glmer* function of the *lme4 R* package [36]. F0 biting time was fitted as a categorical fixed effect, and the random effects fitted were date and an observation-level random effect. For each binomial F2 response the null hypothesis of no association with F0 biting time was tested using a likelihood ratio test. Pairwise differences in F2 biting time proportion between F0 biting times were tested using Wald tests. Predicted F2 biting time proportions with 95% confidence intervals (CI) were calculated from the fitted GLMMs. Bias in predicted proportions and 95% CIs due to Jensen’s inequality [40] was corrected using the approximation of McCulloch et al*.* [37].

## Results

Over 20 nights of sampling, 24,503 wild mosquitoes from local houses adjacent to the PSFS were collected. Of these 28% (n = 6883) were *An. gambiae s.l*. Of the 80% *An. gambiae s.l.* specimens that were successfully amplified by PCR, all were confirmed to be *An. arabiensis*. Of the 5850 F2 *An. arabiensis* (originated from 121 F0 parental *An. arabiensis*) that were released in the PSFS for assay of biting time, 82% were recaptured (52–98% across experimental nights).

**Fig. 1:**
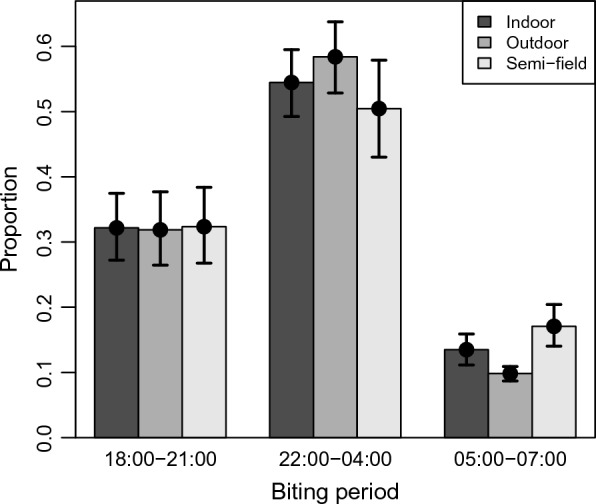
Proportion of *Anopheles arabiensis* biting at different periods of the night (early: 18:00–21:00; mid: 22:00–04:00, late: 05:00–07:00). Colors of the bars. Error bars are 95 CI from fitted model

The biting time pattern of F2 *An. arabiensis* within the PSFS was similar to that observed in the wild population on the same nights (Fig. 1, Table 1); confirming that representative biting behaviours were maintained in the PSFS. In all mosquito collections (made indoors, outdoors or in the PSFS), approximately one third of biting occurred in the early period of the night, two third occurring in the mid period and only one-tenth in the late period.

**Table 1:** Raw data comparison of *Anopheles arabiensis* caught biting at different periods of the night

Biting time	Source of collection	Number caught	% Collected
18:00–21:00	PSFS	1564	32.5
	Indoor	576	31.0
	Outdoor	1183	31.5
22:00–04:00	PSFS	2428	50.4
	Indoor	1046	56.0
	Outdoor	2200	59.0
05:00–07:00	PSFS	821	17.1
	Indoor	252	13.4
	Outdoor	370	10.0

A weak positive correlation of 0.055 was estimated between the biting time phenotypes of F2 and F0 (*t*_*F2-F0*_ [95% CI] = 0.055 [0.001, 0.104]). Using the relationship *h*^*2*^ = 2*t*_*F2-F0*_, this correlation translates to an estimated biting time heritability of 0.110 [0.003, 0.208]. This heritability estimate is sufficiently low that the relative positive bias in the estimate due to assortative mating is likely to be less than 5% (Additional file 3), and therefore should not affect the conclusion that approximately one-tenth of variation in *An. arabiensis* biting time is due to additive genetic variation.

**Fig. 2:**
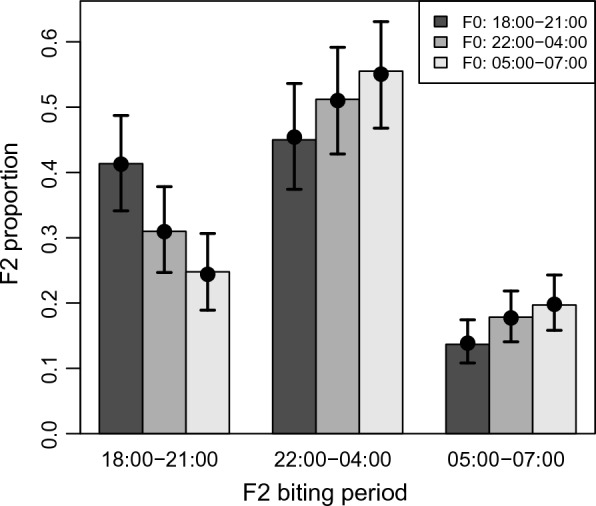
Predicted proportions of F2 biting at different times of the night relative to the biting time of their F0 grandmothers (early: 18:00–21:00 h), mid: 22:00–04:00 h, late: 05:00–07:00 h). Colors of bars denote the biting time of F0 grandmothers. Error bars are 95% CI from the fitted model

There was a significant positive association between F2 biting time (defined as a binary response), and F0 biting time in each of the binomial GLMMs (Table 2). The biting time of early and late biting F2 was positively associated with that of their F0 grandmothers (Fig. 2, Table 2). F2 from early-biting F0 were more likely to bite early than F2 of mid-biting F0 (P < 0.001). F2 from mid-biting F0 were also more likely to bite early than F2 from late-biting F0 (P = 0.018) (Fig. 2, Table 2). A similar positive association was observed for the probability of biting late, with F2 from both mid (P = 0.029) and late-biting (P = 0.001) F0 more likely to bite late than F2 from early-biting F0. However, offspring of mid-biting and late-biting F0 did not differ in their probability of biting late (P = 0.29). Most F2 biting activity occurred in the ‘mid period’ (Fig. 2). The probability of biting in the mid period did not differ between F2 from early and mid F0 (P = 0.066), and F2 from mid and late F0 (P = 0.19). However, F2 of late-biting F0 were more likely to bite in the mid period than those of early-biting F0 (P = 0.002, Fig. 2, Table 2).

**Table 2:** Proportions and 95% confidence intervals (95% CI) of F2 mosquitoes biting at early, mid and late periods of the night, estimated separately by the biting time of their F0 grandmothers

F2 biting time proportion	F0 biting time	F2 predicted biting time proportion (95% CI)	Null hypothesis (H_0_) tests			
			Global H0: early = mid = late	Pairwise H_0_:		
				early = mid	early = late	mid = late
Early/(early + mid + late)	Early	0.41 (0.34, 0.49)	P < 0.0001	P = 0.00061	P < 0.0001	P = 0.018
	Mid	0.31 (0.25, 0.38)				
	Late	0.24 (0.19, 0.31)				
Mid / (early + mid + late)	Early	0.45 (0.37, 0.54)	P = 0.012	P = 0.066	P = 0.0017	P = 0.19
	Mid	0.51 (0.43, 0.59)				
	Late	0.55 (0.47, 0.63)				
Late/(early + mid + late)	Early	0.14 (0.11, 0.17)	P = 0.011	P = 0.029	P = 0.0014	P = 0.29
	Mid	0.18 (0.14, 0.22)				
	Late	0.20 (0.16, 0.24)				

P-values for four null hypothesis tests (H0) are presented: the global null hypothesis that F2 biting time does not vary by F0 biting time, and the three pairwise null hypotheses of equal F2 biting time between early, mid and late F0 biting times. Biting time proportions and p-values were estimated using logit-binomial generalized linear mixed effects models (GLMM; see text for details)

## Discussion

This study, experimentally investigated the heritable genetic basis of biting time tendencies in African malaria vector *An. arabiensis* by comparing phenotypes in a wild F0 population and their F2 offspring under realistic semi-field conditions. The heritability estimate of 0.11 provides evidence that a portion—albeit a minority—of natural variation in biting time is due to additive genetic variation. Heritability estimates in *Drosophila*, the genus where most insect studies of heritability have been performed, range from 0 to 60% [41]. In this context, the estimate of 11% obtained here is relatively low. However, when compared with other behavioural traits alone (as opposed to morphological traits, which tend to have higher heritabilities), this estimate is in the centre of the observed range of 0–20%. Specifically, F2 offspring from early biting F0 grandmothers were more likely to bite earlier than offspring from mid or late biting F0. Similarly, F2 offspring from late biting F0 were more likely to bite later than offspring from early biting F0. In contrast, the offspring of mid and late biting F0 were observed to bite in these two periods with similar frequency. Thus, the likelihood of an F2 feeding within either extreme of the biting activity range (either before 10 pm or after 5am) was associated with their parental phenotype. In general, despite relatively low estimated heritability (11%), this suggests that selection can, therefore, act on biting time of wild African malaria vectors.

This evidence of a genetic association between “extreme” biting time phenotypes (e.g. either early: 18:00–21:00 h, or late 05:00–0.7:00) in offspring and grandparents suggests that the use of ITNs during typical sleeping hours (22:00–05:00 h) could select for shifted biting times in *An. arabiensis* to periods when most people are unprotected. Here deliberately chose to categorize mosquito biting times into periods of unequal length; early (4 h), mid (6 h) and late (3 h), to focus analysis on heritability of behaviours that are specifically problematic for ITNs programmes. The extended length of the ‘mid’ period may account for why most *An. arabiensis* were observed biting during this time regardless of their grandparental biting time phenotype. Finer-scale shifts in mosquito biting time within the mid period may have little epidemiological consequence if people consistently use ITNs while sleeping; which typically occurs between (22:00–04:00) in African communities [27, 42]. In contrast, a shift in mosquito biting to either before people go indoors to sleep (before 10 pm) or after wake up in the morning (5 a.m) would attenuate the impact of ITNs [7]. For example, recent evidence from west Africa found *An. funestus* biting during morning hours (7 a.m–11 a.m) when people are outside in response to widespread use of ITNs [11, 19]. This study show that the tendency of *An. arabiensis* to bite during either of these extreme ranges does have a genetic component; raising concerns that malaria vectors may increasingly adapt their biting times under selection from ITNs or other sources. Such behavioural adaptability represents another potential mechanism of resistance against interventions in malaria vectors in addition to the much more extensively documented physiological insecticides resistance traits. Behavioural resistance through selection of biting times may also contribute to both residual [7] and rebounding of transmission [3].

One previous study also reported evidence of genetic structure between early and late biting *Nyssorhynchus* (*Anopheles*) *darlingi* in the western Amazon [43]. However, a previous investigation of genetic variation between early and late biting *An. arabiensis* in this study area found no evidence of substructuring within a range of candidate circadian genes and single nucleotide polymorphisms [44]. Those authors acknowledged the study had low power to detect genetic variation for biting time due to limitations in sample size and the number of SNPs analyzed [44]; and that a more robust association mapping analysis would be required to be conclusive. The finding of low, but non-zero, heritability and significant association in the biting times between F0 and F2 of *An. arabiensis* suggests that recently observed shifts in malaria vector biting time in response to ITNs [12, 45] may well result from extended evolutionary processes in addition to near-instantaneous phenotypic plasticity.

The study may have some limitations, which could have biased and potentially underestimated heritability. An initial concern was that mosquito biting times observed under semi-field conditions may not be reflective of natural phenotypes. However, this stud confirmed that the nightly pattern of biting activity in the PSFS was similar to that observed in the local wild population on the same nights. Second, ability to detect heritability in biting time may have been reduced because instead of comparing mother–offspring pairs; analysis was based on pooled offspring generated from a cohort of F0 biting in each time period. This design was necessitated in order to produce large enough sample sizes of behavioural bioassays, but prevented identification of variation between individual mosquito mother–offspring pairs to be controlled in the analysis. Thus F2 biting time phenotypes represent an average phenotype from a group of grandmothers, and may mask stronger associations between specific parent–offspring pairs. Third, while a sample size of only 121 F0 may be questionable, it was fairly, sufficient in this study. Despite low heritability estimate, FO-F2 correlation was reliable, based on low P values as described in supplementary information (SI 3). Of concern, biting time was recorded as three categories and not as continuous variable. These could have limited the precision and power of estimate. Fourth, blood feeding F1 at a time corresponding to the capture time of their mothers could have conditioned the mosquitoes, thus generating non-genetic maternal effects [46]. While the existence of maternal effects cannot be ruled out, no evidence of maternal effects was found in related pilot work on mosquito host preference phenotypes *An. arabiensis* (Govella et al. unpublished), nor are aware of any other evidence suggesting maternal effects influence mosquito biting times. Fifth, the age of released mosquitoes ranged between 4 to 10 days over the 20 nights of experimentation. It is possible that some variability could have been introduced due to age-related shifts in biting. However, a previous study of the same species did not detect any variation in biting time with age [47]. Finally, the study was conducted in a single village and single *An. arabiensis* population population, thus limiting generalization of results. Despite these limitations, strong evidence for trans-generational correlation in biting time was detected. It is expected that further investigation under conditions where these potential limitations in design can be addressed would generate a more precise estimate of heritability. In particular, this study highlight the value of crossing experiments between populations of mosquitoes with different biting time phenotypes to determine whether the outcome supports Mendelian pattern of genetic inheritance. This would provide evidence of genetic inheritance of biting time phenotype despite the low heritability obtained in this study.

## Conclusions

This study provides the first experimental confirmation of the existence and magnitude of heritability in the biting time of a major African malaria vector. These results lay a foundation for further investigation of the genetic basis of this phenotype and other mosquito feeding behaviours (host preference and location of biting) whose evolution in response to interventions could undermine malaria elimination efforts in Africa. These results suggest that although *An. arabiensis* biting time may be primarily driven by non-genetic factors, this behavioural phenotype may also be partly heritable. Therefore, the observed changes in biting time in field populations might be influenced by longer-term selection arising from intervention in addition to the likely larger role of non-genetic phenotypic plasticity. Regardless of the relative contribution of each of these processes, the increasing evidence of shifts in biting times in malaria vector populations highlight the urgent need for complementary interventions that can target mosquitoes in outdoor environments and outside of typical sleeping hours.

### Supplementary Information

**Additional file 1.** Portable semi-field system.

**Additional file 2.** Protocol and experimental design for assays of heritability in biting time.

**Additional file 3.** Statistical methods for estimating the heritability of biting time.

**Additional file 4.** R-codes showing how assortative mating influence the phenotypic correlation among F1 mates and consequent expected bias in estimation of heritability.

## Data Availability

Access and use of data supporting this article are available in publicly repository (https://doi.org/10.5061/dryad.0vt4b8gzv).
