# Supplementary material for: Heritability of biting time behaviours in the major African malaria vector Anopheles arabiensis
Source: Malar J. 2023 Aug 16;22:238. doi: 10.1186/s12936-023-04671-7 (PMC10433675; doi:10.1186/s12936-023-04671-7)
Supplement: Supplementary file 2 — Additional file 2. Protocol and experimental design for assays of heritability in biting time. [file 12936_2023_4671_MOESM2_ESM.docx]

**Protocol and Experimental design for bioassays of heritability in biting time**

In Lupiro village, female *An. arabiensis* were collected host seeking at different times of the night using Human Landing Catches (HLC). The collections were conducted from peridomestic area at around 4 houses. With HLC, a volunteer exposes his lower limbs and aspirates mosquitoes landing on his exposed limbs. Volunteers collected mosquitoes host seeking between 18:00–07:00hrs for two consecutive nights (14^th^ and 15^th^ of July 2015) at 4 houses. Mosquitoes visually identified as belonging to *An. gambiae s.l.* (Coetzee 2020 Malar J *19:70*) were grouped into one of 3 biting time periods on the basis of their collection time: early biting (18:00-21:00), mid biting (22:00-04:00), and late biting (05:00-07:00) and were placed in separate holding cages (0.36 m x 0.39 m). Biting time activity was broken into these 3 discrete categories to correspond with times when people are likely to be either indoors and protected by ITNs (‘mid’), or outdoors and unprotected (early and late). Cages were maintained under ambient conditions within the PSFS. Of the 245 female *An. gambiae s.l.* obtained, 218 survived the transition into cages (71, 98 and 49 from early, mid and late biting groups respectively). These mosquitoes were provided with a blood meal via arm feeding for two days consecutively post capture, with each group blood fed in the same time period they were collected. After blood feedings, captured mosquitoes were individually placed into small, separate cages (0.15 m × 0.17 m) inside PSFS, and assigned a unique identification code (ID). Each small cage contained a petri dish lined with wet cotton and filter paper on top as an oviposition substrate. A total of 142 individual mothers successful laid eggs with the rest either flushed the blood or died before oviposition. After oviposition, each individual mother was killed and stored on silica gel in a labeled 1.5 ml eppendorf tube and identified to species level by polymerase chain reaction (Scott et al 1993 *Am J Trop Med Hyg 49: 520-529*). Of 142, 121 individual mothers (43, 51 and 27 from early, mid and late respectively) confirmed to be *An. arabiensis*, and these formed the parental generation (F0) for experiment in biting time heritability. The eggs from each confirmed *An. arabiensis* mother were transferred into a small water-filled bowl (~0.1 m diameter) for hatching and subsequent larval development. Each bowl was labelled correspondingly to the ID of their individual mothers. The larvae were fed on Tetramin fish food (Tetra, Melle, Germany). Pupae emerging from each egg clutch were combined into groups on the basis of the biting time category of their mother, and transferred into a larger holding cage for emergence (separate cages for each biting time phenotype). The first filial generation (F1) adults emerging from these pupae were maintained on 10% glucose solution for up to 5 nights, and then given a blood meal via arm-feeding as described for the parental generation. This process yielded a second generation (F2) for use in bioassays. Experiments were conducted on the F2 generation to increase the sample size in each biting time phenotype.

Heritability was tested using F2 *An. arabiensis* that were released in a PSFS and recaptured using HLC to measure their biting time phenotype, and compare it with that of their F0 grandmothers. Bioassays were conducted over 20 consecutive nights. One each night of bioassays, 300 F2 *An. arabiensis* (100 from each biting time phenotype group) were released into the PSFS all at the same time at 17:00 hours in the evening, with the exception of one trial night in which only 50 of each phenotype were available. The age of released mosquitoes ranged between 4 to 10 days old representing a mix of young and old mosquitoes (Milali et al 2019 PLoS ONE 14(8): e0209451). Prior release, F2 mosquitoes were marked at 15:00 hours with either red, yellow or blue fluorescent dust colors according to their grandmothers’ biting time phenotype (18:00-21:00, 22:00-04:00, and 05:00-07:00). No evidence exists for the powder to affect the biting time activity of mosquitoes, however, as precaution, we allowed atleast three hrs rest within a cup before release for mosquitoes to clear up the dust themselves within a cup. They remained starved from 15:00 hours until the time of release at 17:00 hours. All marked mosquitoes were released simultaneously at the center within the PSFS. A volunteer entered the PSFS to conduct mosquito collections by HLC from 18:00 to 07:00 hrs. This way, mosquitoes had atleast one hrs to orient themselves within the PSFS before feeding activity start. All mosquitoes that were attempting to feed during each hour were collected and grouped into a common holding cup. In the morning after collection, mosquitoes captured during each of the 3 biting periods were identified and recorded based on their dust color to their grandmother’s biting time. Thus both the biting time period of each individual could be linked to that of their grandmother. The morning end of each experimental night, thorough search and with back-pack aspiration of mosquitoes on the floor, roofs and side walls within the PSFS was conducted. Searching for unrecaptured mosquitoes was conducted for 10-15 mins. This was to ensure mosquitoes captured in the next night experiment were not from the group released the night before. Additional HLC collections were conducted at 2 local houses adjacent to the PSFS within approximately 40m away on the same nights as bioassays to assess whether the pattern of biting activity in the PSFS was consistent with that of the wild population. Nearby houses from the PSFS were chosen so as to try maintaining similar environmental effects (for example winds, temperature and lights) between field and in the PSFS, which might affect the behavioural outcomes of the mosquitoes (Kampango et al 2011 Med Vet Entomol 25:240-246). Wild mosquitoes were collected from inside and outside local houses. In the morning after night collections, captured mosquitoes were killed using ethanol, sorted, identified to morphological level as *An. gambies s.l.* and their numbers recorded according to the time of their capture. All Individuals mosquitoes of *An. gambiae s.l.* (field collected mosquitoes) were stored in the eppendorf tube for sibling species identification by PCR (Scott et al 1993 Am J Trop Med Hyg 49: 520-529.)
