# Supplementary material for: Heritability of biting time behaviours in the major African malaria vector Anopheles arabiensis
Source: Malar J. 2023 Aug 16;22:238. doi: 10.1186/s12936-023-04671-7 (PMC10433675; doi:10.1186/s12936-023-04671-7)
Supplement: Supplementary file 3 — Additional file 3. Statistical methods for estimating the heritability of biting time. [file 12936_2023_4671_MOESM3_ESM.doc]

Supplementary methods for estimating the heritability of biting time

*Background*

Narrow sense heritability of biting time, *h^2^*, was estimated from the correlation between grand-offspring (F2) biting time and grandparental (F0) biting time, *t_F2-F0_*. This correlation coefficient was estimated using a mixed-effects ordinal probit regression model implemented using the *MCMCglmm* function in the *MCMCglmm* package (Hadfield 2020 Journal of statistical Software, 33: 1-22) for *R* version 4.0.2. This approach of modelling a discrete trait as the manifestation of an underlying continuous “liability” (here the tendency towards biting at a specific time) and estimating heritability on the liability scale is standard in quantitative genetics (de Villemereuil et al. 2016 Genetics 204:1282-1294).

*Estimation of the F2-F0 correlation*

F0 and F2 biting time, which is an ordered categorical response (early < mid < late), was modelled as resulting from trichotomizing a latent continuous biting time scale assumed to have a standard normal distribution (having zero mean and unit variance) around two threshold parameters represented by two intercepts in the regression model. Each experiment was conducted by releasing three batches (i.e. early, mid and late biting F2) of mosquitoes on each of 20 days followed by HLC collection of biting mosquitoes by two volunteers. Temperature was recorded on each day. To adjust for potential variation in mean biting time by generation (F0 and F2), temperature, between two HLC volunteers, or over time, the GLMM included fixed effects of generation, temperature (separately for each generation), volunteer, and a natural cubic spline with three degrees of freedom for day of the experiment. Continuous variables (temperature and day) were scaled to have zero mean and unit variance. Variation in biting time among batches (each batch consisting of a single F0 and her F2 granddaughters) was modelled by a random intercept with variance *V_b_*. Variation between generations within batches was modelled by a random effect with variance *V_g:b_*. The correlation between F2 and their F0 grandmothers is $t_{F2-F0}=\frac{V_{b}}{V_{b}+V_{g:b}+1}$. The 1 added to the total variance in the denominator comes from the probit link. Intuitively, this correlation will be high when variation between batches (*V_b_*) is high relative to the sum of the intergenerational variance and 1 (*V_g:b_* + 1). Conversely, when intergenerational variance is high relative to inter-batch variance (i.e. F2 do not bite at similar times to their F0 grandmothers), then the correlation coefficient will be low. Because of the inclusion of the fixed effects, this correlation can be considered to be adjusted for the effects of generation, temperature, volunteer and date. Estimates of the fixed and random effect parameters are given in the Table 1.

**Table 2.** Summary statistics from *MCMCglmm* for the model used to estimate the inter-batch and inter-generational variances. biting.time: ordinal biting time; gen.num: indicator for generation, where 0 indicates F0 and 1 indicates F2; temp: temperature in degrees Celsius; volunteer: the HLC volunteer; date: days since the start of the experiment; batch: random intercept for batch; gen:batch: random intercept for variation between generations within batches; post.mean: posterior mean; eff.samp: effective number of MCMC samples.

Iterations = 120001:519601

Thinning interval = 400

Sample size = 1000

DIC: 9534.102

G-structure: ~batch

post.mean l-95% CI u-95% CI eff.samp

batch 0.0598 1.335e-05 0.1062 1000

~gen:batch

post.mean l-95% CI u-95% CI eff.samp

gen:batch 0.02205 2.775e-09 0.07208 1000

R-structure: ~units

post.mean l-95% CI u-95% CI eff.samp

units 1 1 1 0

Location effects: biting.time ~ I(gen.num - 0.5) + I(scale(temp * (gen.num == 0))) + I(scale(temp * (gen.num == 1))) + volunteer + ns(scale(date), df = 3)

post.mean l-95% CI u-95% CI eff.samp pMCMC

(Intercept) 7.94746 5.88352 10.41172 1000 <0.001 ***

I(gen.num - 0.5) -15.04256 -19.87721 -10.60614 1000 <0.001 ***

I(scale(temp * (gen.num == 0))) -1.75570 -2.29585 -1.23689 1000 <0.001 ***

I(scale(temp * (gen.num == 1))) -0.13841 -0.24858 -0.04278 1000 0.006 **

volunteerTS -0.06164 -0.24458 0.12932 1000 0.514

ns(scale(date), df = 3)1 -0.17170 -0.57622 0.17383 1000 0.370

ns(scale(date), df = 3)2 -0.17179 -0.72201 0.41138 1000 0.550

ns(scale(date), df = 3)3 0.12718 -0.20644 0.48752 1000 0.454

---

Signif. codes: 0 ‘***’ 0.001 ‘**’ 0.01 ‘*’ 0.05 ‘.’ 0.1 ‘ ’ 1

Cutpoints:

post.mean l-95% CI u-95% CI eff.samp

cutpoint.traitbiting.time.1 1.468 1.424 1.523 1000

*Estimation of heritability*

Assuming random mating, *h^2^* = *t_F2-F0_*/*r*, where r is the coefficient of relatedness. The coefficient of relatedness between a single F0 grandmother and her F2 granddaughter is 0.25, giving *h^2^* = 4*t_F2-F0_*, but the F2-F0 correlation was contributed to by two grandmothers, giving *h^2^* = 2*t_F2-F0_* (Falconer et al, 1996 4^th^ edition, harlow: Addison Wesley Longman). However, this relationship applies only under random mating. Where mating is assortative, the phenotypic correlation between relatives should be accounted for, or the estimate of *h^2^* will be biased upwards (Nagylaki 1978 Ann Human Genetics 42:131) . The degree of phenotypic correlation in this study is complex, and its potential impact on estimation of *h^2^* is discussed in the next section.

*Bias in estimated heritability due to assortative mating in the F1 generation*

In the F0 generation, grandmothers and grandfathers can be assumed to have mated at random. In the F1 generation, mating can be assumed to have been random, but within groups separated by grandmaternal biting time, which will have induced a phenotypic correlation that depends on *h^2^*. The effect of assortative mating is to increase the expected F2-F0 correlation so that in order to avoid a positive bias in estimation of *h^2^*, it should be divided by a factor of (1 + *r_P_*)(1 + *r_P_h^2^*), where *r_P_* is the phenotypic correlation between F1 mates (Nagylaki 1978 Ann Human Genetics 42:131). For example, for *r_P_* = 0.25 and a heritability of 0.7, a heritability that was estimated without accounting for assortative mating, as *h^2^* = 2*t_F2-F0_*, would be overestimated by 47%. We do not know the degree of assortative mating in this study, and therefore cannot adjust for it, but using simulations we show that *r_P_* is unlikely to be greater than 0.1 for *h^2^* < 0.5 (see below), in which case the positive bias in *h^2^* would be < 16%. We therefore estimated heritability using *h^2^* = 2*t_F2-F0_* but with the caveat that the low heritability estimates (< 0.5) are likely to be slightly positively biased and high (> 0.5) estimates could be severely positively biased due to deviation from random mating. In the next section we explore the likely extent of this bias in our biting time data.

*Estimation of bias in heritability due to assortative mating using simulated data*

We simulated biting time data of similar size and structure to the mosquito biting time data to explore how assortative mating influences the phenotypic correlation among F1 mates, and the consequent expected bias in estimation of heritability. 10,000 data sets were simulated across the full range of heritability values from zero to one. Grandparental biting time was simulated as an integer score, as in the data analysis. For computational speed, F1 biting time was simulated and modelled as a continuous directly measured scale rather than an ordinal manifestation of a liability scale. Also for computational speed, we avoided the need for random effects by simulating only a single pair of F1 offspring from each of 20 pairs of F0 grandmothers per biting time category (n = 60 F1 pairs). Assortative mating was simulated between F1 males and females by assuming random mating within the three groups defined by the F0 biting time of their grandmothers. To illustrate the effect of assortative mating, three outcome measures were plotted against true *h^2^*: the estimated phenotypic correlation (*r_P_*); the biased estimate of *h^2^*; and bias in estimating *h^2^* (Fig. 1-3). The smoothed relationship (i.e. averaging over sampling error in *r_P_*) between each outcome and true *h^2^* was estimated using OLS regression with a natural cubic spline with four degrees of freedom fitted to true *h^2^*. Full details of the simulation methods are given in the R code provided (**SI 4**).

The estimated phenotypic correlation among F1 mates was low (*r_P_* < 0.1) for *h^2^* < 0.5, rising to 0.5 as *h^2^* approached its maximum value of one (Fig. 1). Estimated *h^2^* including the expected bias due to not accounting for assortative mating was calculated as *h^2^*(1 + *r_P_*)(1 + *r_P_h^2^*) (Fig. 2), and percentage bias was calculated as 100 × ([biased *h^2^*] – [true *h^2^*])/[true *h^2^*] (Fig. 3). As expected, bias was low to moderate (0-17%) for *h^2^* < 0.5, but rose steeply (17-123%) for *h^2^* > 0.5. Estimated *h^2^* exceeded the maximum possible *h^2^* value of one for true *h^2^* > 0.7.

We conclude that estimates of biting time *h^2^* that are close to and above 0.5 could be moderately to severely positively biased by not accounting for the unknown degree of assortative mating, but that bias in estimates of *h^2^* below 0.5 is likely to be low (< 17%), and bias in *h^2^* estimates below 0.3 is likely to be negligible (< 4%).
