# Supplementary material for: Heritability of biting time behaviours in the major African malaria vector Anopheles arabiensis
Source: Malar J. 2023 Aug 16;22:238. doi: 10.1186/s12936-023-04671-7 (PMC10433675; doi:10.1186/s12936-023-04671-7)
Supplement: Supplementary file 4 — Additional file 4. R-codes showing how assortative mating influence the phenotypic correlation among F1 mates and consequent expected bias in estimation of heritability. [file 12936_2023_4671_MOESM4_ESM.pdf]

Simulate.F1.mate.correlation.2020-11-22.R

pauljohnson

2020-11-23

R script to support the calculation of heritability of mosquito biting time.

Background
The aim of this script is to simulate data similar in size and structure to the mosquito biting time data, to show how assortative mating influences the phenotypic correlation among F1 mates, and the consequent expected bias in estimation of heritability.

Start script
Save figures to directory:

```
knitr::opts_chunk$set(fig.path = "Simulate.F1.mate.correlation.2020-11-22_figures/")
```

Load packages:

```
library(splines)
library(scales)
```

Clear memory:

```
rm(list = ls())
```

Simulation options
Simulate assortative mating among F1 females and males? (change this to illustrate lack of bias when mating is not assortative).

```
assort <- TRUE
```

Number of offspring (similar to mosquito experiment):

```
n <- 60
```

Choose heritability values Simulate h2 values across its range from 0 to 1.

```
n.sim <- 10000
h2.list <- seq(0, 1 - 1/n.sim, length.out = n.sim) + 0.5/n.sim
```

Set random seed for repeatability https://www.random.org/integers/?num=1&min=0&max=1000000000&col=1&base=10&format=html&rnd=new

```
set.seed(567952879) # Timestamp: 2020-11-23 11:13:18 UTC
```

Loop over h2 values estimating phenotypic correlation among F1 mates

```
rP <-
  sapply(h2.list, function(h2) {

    # variance parameters
    Vp <- 1 # phenotypic variance
    Va <- Vp * h2 # genetic variance
    Ve <- Vp - Va # environmental/error variance
```

```r
  # start data frame to store values of relatives
  dat <- data.frame(id = 1:n)

  # simulate trait values (biting time) of grandparents (F0), and scale to standard normal
  dat$GMm <- as.vector(scale(sample(rep(1:3, length.out = n)))) # mothers of mothers
  dat$GMf <- as.vector(scale(sample(rep(1:3, length.out = n)))) # mothers of fathers
  dat$GFm <- as.vector(scale(sample(rep(1:3, length.out = n)))) # fathers of mothers
  dat$GFf <- as.vector(scale(sample(rep(1:3, length.out = n)))) # fathers of fathers

  # make grandmothers perfectly correlated, as in the mosquito experiment
  # (this doesn't affect the h2 estimate)
  if(assort) dat$GMf <- dat$GMm

  # simulate F1 values as the mean of their parents * h2 + environmental noise
  dat$F1m <- h2*(dat$GMm + dat$GFm)/2 + rnorm(n, sd = sqrt(Ve))
  dat$F1f <- h2*(dat$GMf + dat$GFf)/2 + rnorm(n, sd = sqrt(Ve))
  var(dat$F1m)
  var(dat$F1f)

  # these lines can be run if stepping into the loop to show that
  # the simulated h2 values are accurately estimated:
  if(FALSE) {
    # estimate h2 by regression of F1 males on F0
    fit <- lm(F1m ~ I((GMm + GFm)/2), data = dat)
    c(h2 = h2, h2.est = as.vector(coef(fit)[2]))

    # estimate h2 by regression of F1 females on F0
    fit <- lm(F1f ~ I((GMf + GFf)/2), data = dat)
    c(h2 = h2, h2.est = as.vector(coef(fit)[2]))

    # ...these estimates can be shown to be accurate by choosing very large n, e.g. 10000
  }

  # permute F1 fathers to avoid correlations between F1 parents within F0 biting time
  # groups (i.e. stratified by F0 grandmother's phenotype). thus the only correlation is
  # due to stratification.
  dat$F1f.mix.i[dat$GMf < -1] <- sample(dat$id[dat$GMf < -1])
  dat$F1f.mix.i[dat$GMf > 1] <- sample(dat$id[dat$GMf > 1])
  dat$F1f.mix.i[is.na(dat$F1f.mix.i)] <- sample(dat$id[is.na(dat$F1f.mix.i)])

  # if no assortative mating then uncorrelate F1f from F1m
  if(!assort) dat$F1f.mix.i <- sample(dat$F1f.mix.i)

  # re-order F1f and GMf so that they are correctly aligned with offspring
  dat$F1f.mix <- dat$F1f[dat$F1f.mix.i]
  dat$GMf.mix <- dat$GMf[dat$F1f.mix.i]

  # correlation between F1 mates (rP)
  cor(dat$F1f.mix, dat$F1m)
})
```

Plot results

Function to plot a smooth spline (and optionally print a predicted value)

```
lines.spline <-
  function(x, y, dof = 4, pred.x = NULL) {
    require(splines)
    fit <- lm(y ~ ns(x, df = dof))
    x.pred <- seq(min(x), max(x), length.out = 100)
    lines(x.pred, predict(fit, data.frame(x = x.pred)), lty = 2, lwd = 1.5)
    if(!is.null(pred.x)) {
      return(data.frame(x = pred.x, y = predict(fit, data.frame(x=pred.x))))
      }
  }
```

Set up plotting parameters

```
col <- alpha("blue", 0.1)
pch <- 16
```

Plot rP against h2
Add spline

```
plot(h2.list, rP, xlab = expression(h^2), ylab = expression(r[P]), col = col, pch = pch)
lines.spline(x = h2.list, y = rP)
abline(h = 0)
title(expression("Fig. 1. Phenotypic correlation among F1 mates, r"[P]*", by heritability, h"^2))
```

Fig. 1. Phenotypic correlation among F1 mates, r_P, by heritability, h^2

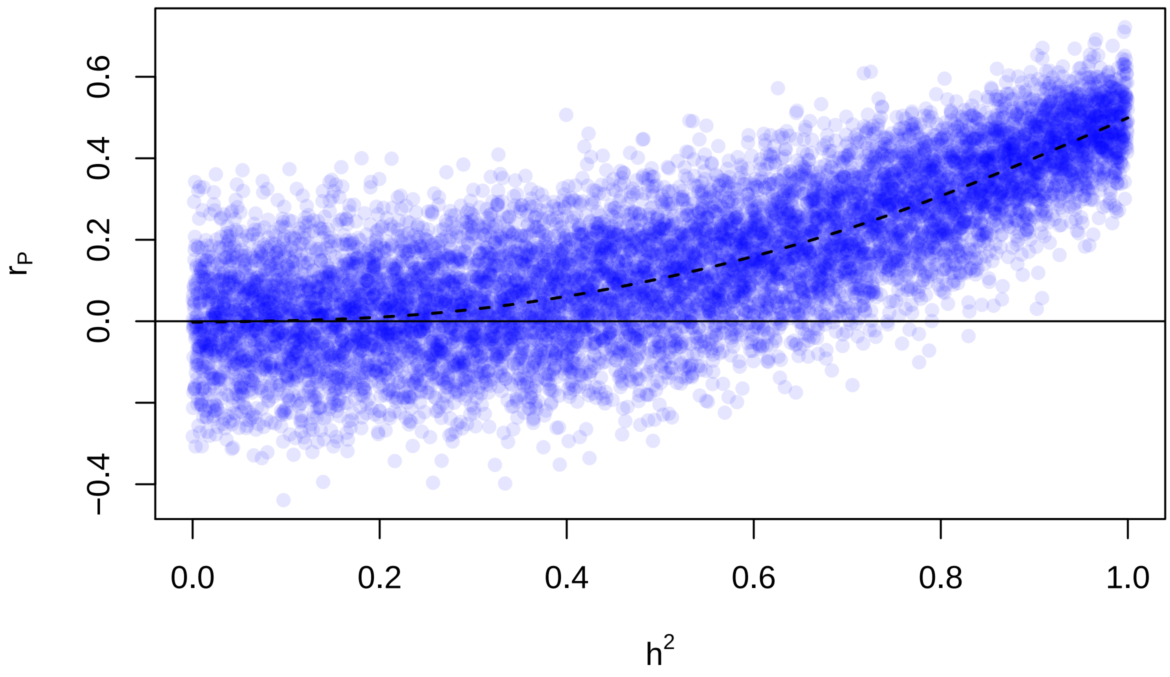

Plot h2 with expected bias against true h2
Add spline

```
h2.with.bias <- h2.list * (1 + rP) * (1 + rP * h2.list)
plot(h2.list, h2.with.bias, xlab = expression(h^2),
     ylab = "",
     col = col, pch = pch)
```

```
mtext(expression("Biased h"^2*": "*h^2 * (1 + r[P]) * (1 + r[P] * h^2)), side = 2, line = 2.5)
lines.spline(h2.list, h2.with.bias)
abline(0, 1, lty = 3)
title(expression("Fig. 2. Heritability, h"^2*", with expected bias due to r"[P]>0*", by true h"^2))
legend("topleft", legend = c("Biased", "Unbiased"), lty = 2:3)
```

Fig. 2. Heritability, h^2, with expected bias due to $r_P > 0$, by true h^2

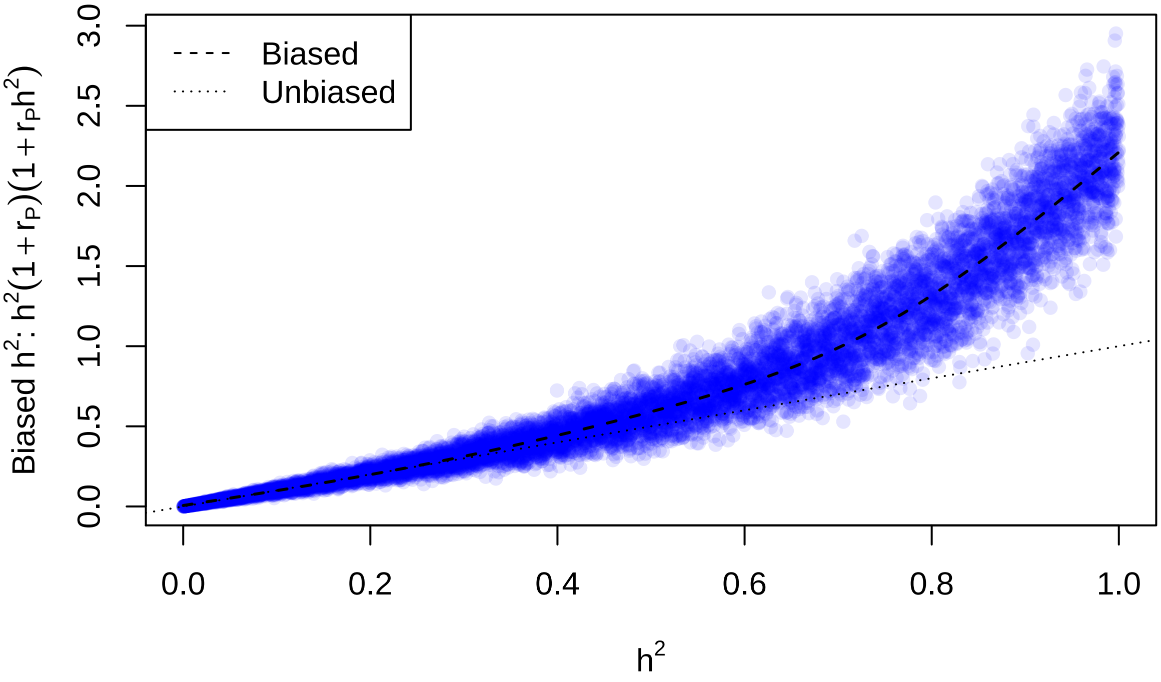

Plot percentage bias in h2 (given rP) against h2
Add spline and output expected bias in h2 at h2=0.5

```
h2.percent.bias <- 100 * (h2.with.bias/h2.list - 1)
plot(h2.list, h2.percent.bias, xlab = expression(h^2),
     ylab = expression("% bias in h"^2),
     col = col, pch = pch)
title(expression("Fig. 3. Bias in h"^2*" due to r"[P]>0*", by true h"^2))
abline(h = 0)
h2.percent.bias.at.h2 <-
  lines.spline(h2.list, h2.percent.bias, pred.x = seq(0, 1, by = 0.1))
```

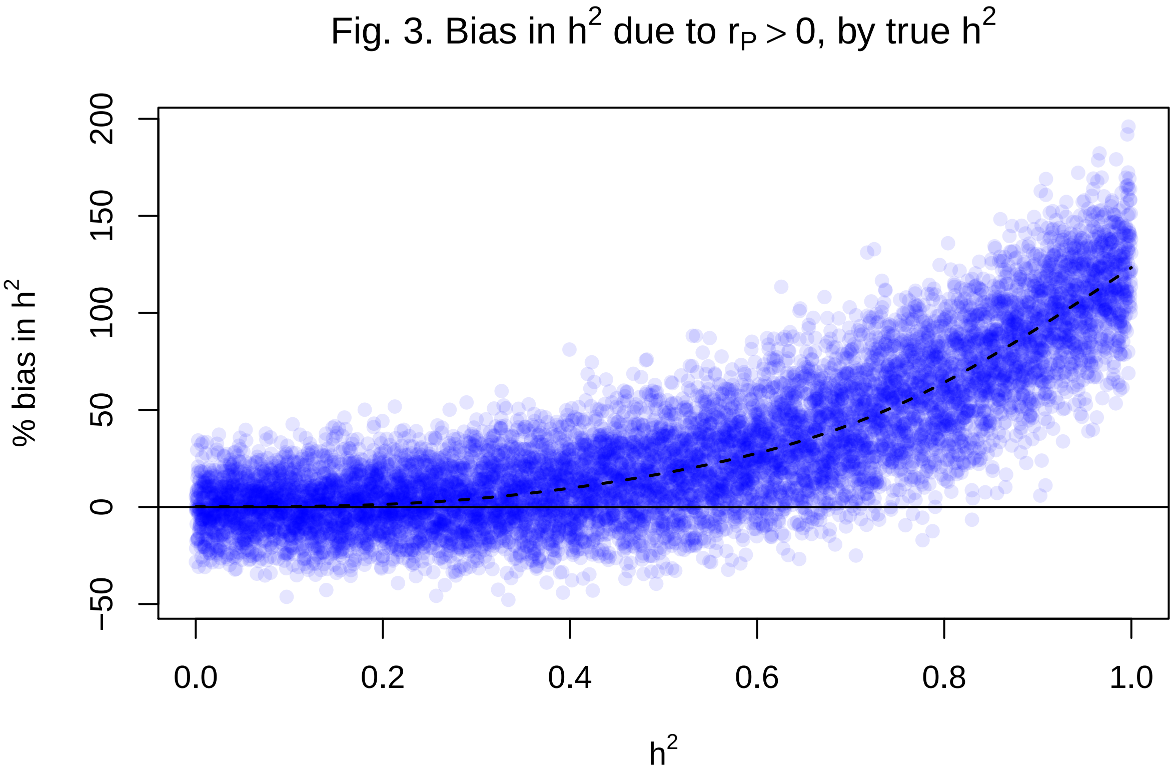


```r
names(h2.percent.bias.at.h2) <- c("h2", "percent.bias.in.h2.estimate")
h2.percent.bias.at.h2$percent.bias.in.h2.estimate <-
  round(h2.percent.bias.at.h2$percent.bias.in.h2.estimate)
```

Expected percentage bias in h2 when h2 = 0.3-0.5:

```r
h2.percent.bias.at.h2
```

```
##     h2 percent.bias.in.h2.estimate
## 1  0.0                           0
## 2  0.1                           0
## 3  0.2                           1
## 4  0.3                           4
## 5  0.4                          10
## 6  0.5                          17
## 7  0.6                          28
## 8  0.7                          43
## 9  0.8                          64
## 10 0.9                          92
## 11 1.0                         123
```
